# Pleural effusion in 11:14 translocation q1 multiple myeloma in the setting of proteasome inhibitor presents therapeutic complexity

**DOI:** 10.1007/s12254-018-0388-y

**Published:** 2018-02-21

**Authors:** Malik Ghannam, Maria Bryan, Erik Kuross, Brent Berry

**Affiliations:** 1 Neurology Department, University of Minnesota, Minneapolis, MN USA; 2 University of Minnesota, Minneapolis, MN USA; 3 Internal Medicine Department, Hennepin County Medical Center, Minneapolis, MN USA

**Keywords:** Primary myelomatous pleural effusion, Multiple relapses of MM, Poor prognosis, Rare entity, Proteasome inhibitors and lung damage, Case report

## Abstract

**Background:**

Primary malignant pleural effusion has been reported in about 134 cases of multiple myeloma (MM). Associated pleural effusions in cases of MM portend a poor prognosis and identifying them is highly relevant. Reported is the case of a man diagnosed with MM who developed primary myelomatous pleural effusion in the setting of multiple relapses and subsequent mortality within 2 months of the pleural effusion diagnosis.

**Presentation:**

A 61-year-old African American man was diagnosed with MM in 2011. He received induction therapy of lenalidomide and dexamethasone and an autologous stem cell transplant in 2012. Over the next 5 years, the patient went through alternating periods of remission and relapse that were treated with two rounds of thoracic spine radiation therapy and chemotherapeutic agents. In September 2017, the patient presented with worsening dyspnea and was found to have pleural effusion. Fluid analysis showed plasma cell dyscrasia. Fluid drainage was performed, then the patient was discharged after 1 week which was followed by rapid re-accumulation of fluid and rehospitalization about 10 days after discharge. The patient passed away a few weeks after the second admission.

**Conclusion:**

Pleural effusion carries a differential diagnosis which may include malignancy but is commonly thought to be less specific to multiple myeloma but should still remain in the differential diagnosis. To our knowledge, this is the first case of myelomatous pleural effusion (MPE) that was reported after multiple relapses of MM. MPE is a very rare complication of MM, and its presence is a strong indicator of imminent mortality and need for comfort care in case of multiple relapses. End-stage pleural effusion in MM in the setting of proteasome inhibitor adds more therapeutic and diagnostic challenges.

## Introduction

Multiple myeloma (MM) is a clonal plasma cell neoplasm accounting for 1% of all malignancies in the United States and 10% of hematological malignancies [1]. The current literature continues to describe a few cases of infiltrative myelomatous pleural effusions. In 2005, primary malignant pleural effusion associated with MM was reported in about 80 cases [2], whereby another 50 cases of MPE in MM have since been reported in the literature. Pleural effusions can be seen in MM but few are found to be malignant, perhaps less than 1% [3]. Associated pleural effusions in cases of MM portend a poor prognosis and identifying them is highly relevant [4]. Here, the case of a middle to late aged man with kappa light chain MM disease diagnosed in 2011 presenting to the hospital with worsening dyspnea secondary to pleural effusion is described.

## Case presentation

A 61-year-old man presented in August 2011 with severe upper back pain and was found to have a T5 compression fracture with focal lytic lesions. The patient had acute renal failure, anemia, hypercalcemia, and a bone marrow biopsy suspicious for MM. Immunohistochemistry showed a neoplastic plasma cell population with diminished CD45 expression representing approximately 19% of the CD45-positive cell population. The cells also showed coexpression of CD38, CD138, and CD56 but were negative for CD19 and CD20. Furthermore, interphase FISH also documented the presence of an 11;14 rearrangement in approximately 33% of interphase nuclei. At this time, the patient was ISS and R‑ISS stage II with an LDH of 203 IU/L, albumin of 3.5 g/dL, beta-2-microglobulin of 4 mg/L, and no high-risk translocations.

Patient underwent thoracic spine radiation therapy in August 2011 and began induction therapy with five 28 day cycles of linalidomide/dexamethasone. The cycles consisted of 21 days of 25 mg Linalidomide followed by a 7 day break with weekly doses of 40 mg dexamethasone. This was followed by high-dose chemotherapy and an autologous stem cell transplant in February 2012. Subsequent biopsy showed complete remission and the patient declined a second stem cell transplant. He was placed on a daily oral 10 mg maintenance dose of lenalidomide. In May 2012 his myeloma lab studies showed 0.1 g monoclonal spike (M spike). Maintenance lenalidomide was reduced to 5 mg due to myelosuppression and temporarily discontinued due to herpes zoster infection in November 2012. In January 2013, at the patient’s one year transplant follow-up, bone marrow biopsy showed subtle relapse of disease. On immunohistochemical staining, the kappa to lambda ratio was 2.2:1 so FISH was suggested to correlate. FISH showed an 11;14 translocation in 14% of interphase cells consistent with bone marrow involvement by the patient’s plasma cell myeloma as characterized by FISH in his previous study. Subsequently lenalidomide was increased to 10 mg each dose.

In March 2013, the patient experienced increasing neck pain with labs showing increased kappa free light chain and magnetic resonance image (MRI) showed new cervical spine lytic lesions. Bortezomib and dexamethasone were empirically started. The patient’s kappa free light chain decreased immediately, and his neck pain improved. The bone marrow biopsy after bortezomib and dexamethasone therapy showed approximately 5–10% bone marrow involvement by fibrosis and only scattered plasma cells. In addition, FISH testing showed only 3% of interphase nuclei having the 11;14 translocation. The patient’s bortezomib and dexamethasone therapy were reduced to weekly treatments due to neuropathy complications and were discontinued altogether after June 2013 due to intractable neuropathy.

In September of 2013, the patient was found to have recurrence of his MM with 50% bone marrow involvement, increased lytic bone lesions and hypercalcemia. The patient was put on 25 mg lenalidomide and dexamethasone therapy for 21 days for a total of thirteen cycles from October 2013 to October 2014. Bone marrow biopsy in October 2014 showed 11–12% polyclonal plasma cells without definitive morphologic or immunophenotypic evidence of recurrent plasma cell myeloma, and the patient was transitioned to maintenance therapy of lenalidomide 10 mg daily. The patient became neutropenic and therapy was withheld in November 2014. In December 2014 bone marrow biopsy showed recurrent MM. Further FISH analysis detected an 11;14 translocation in 36% of the interphase nuclei as well as an extra copy of 1q in 39% of interphase nuclei. This amplification of 1q was not present in the patient’s original FISH analysis back in 2011 suggesting cytogenetic evolution of the patient’s clone. He began pomalidomide 4 mg for one month with dexamethasone 40 mg weekly. Subsequent bone marrow biopsy in September 2015 results indicated disease progression.

**Fig. 1:**
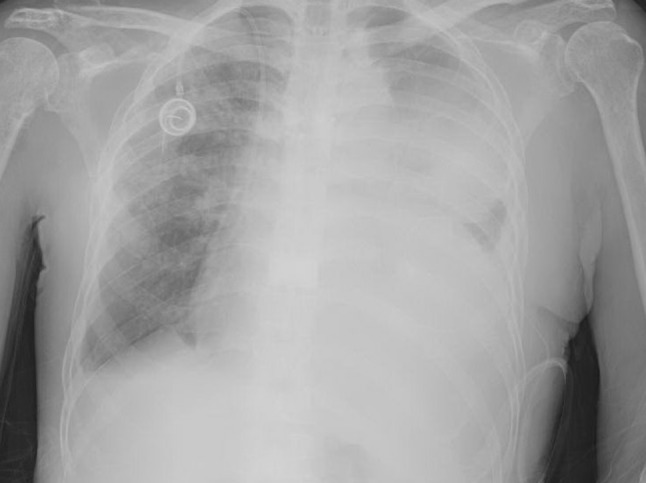
Chest X‑ray with large left malignant pleural effusion immediately after pigtail catheter placement but before fluid drainage

**Fig. 2:**
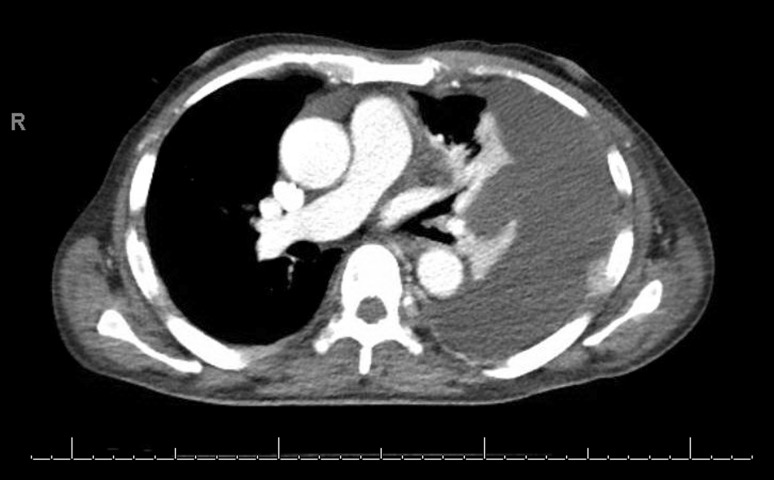
Chest computed tomography (CT) with intravenous contrast demonstrating malignant left pleural effusion with left lower lobe collapse and segmental atelectasis in left upper lobe

In October 2015, the patient started carfilzomib, pomalidomide, dexamethasone combined treatment; this was done for 5 cycles at which point the patient went for bone marrow transplant evaluation and was placed on a donor list. In March, 2016 it was decided to give the patient a break from chemotherapy as he had been stable in partial remission and in an effort to help his pancytopenia. Unfortunately, a whole body MRI on 26 April 2016 showed progression of diffusely infiltrative MM so he was started on daratumumab with Linalidomide. The disease continued to progress and the patient’s regimen was changed to elotuzumab/Linalidomide/dexamethasone on 30 December 2016. During this time the patient was admitted to the hospital with acute kidney injury but imaging was not suspicious for pleural effusion. Continued progression forced the switch to NINLARO/dexamethasone and then carfilzomeb/thalidomide/dexamethasone. It was then in September 2017 that the patient presented to the hospital with dyspnea and was found to have bilateral pleural effusion on chest radiography (Fig. 1). His chest computed tomography (CT) was significant for left lower lobe collapse with basilar opacities as well as patchy ground glass opacities and interlobular septal thickening (Fig. 2).

**Fig. 3:**
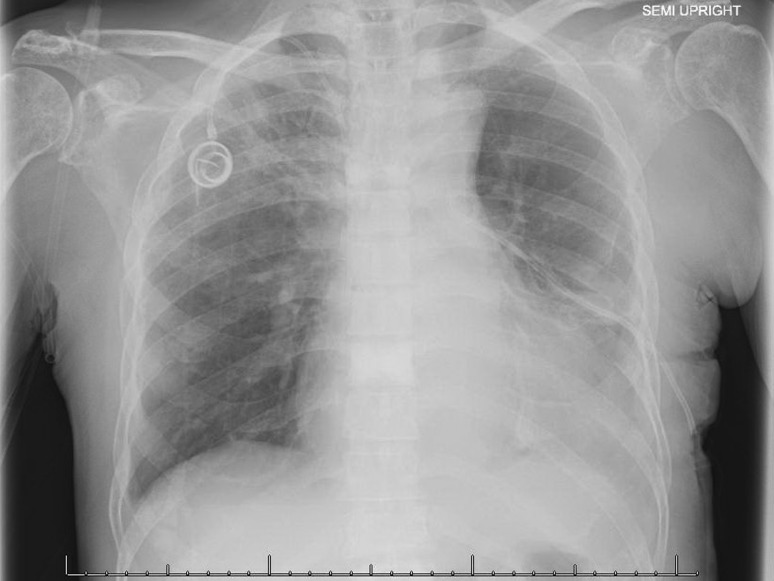
Chest X‑ray post placement of left hemithorax pigtail catheter with drainage and significant decrease in large left pleural effusion

**Fig. 4:**
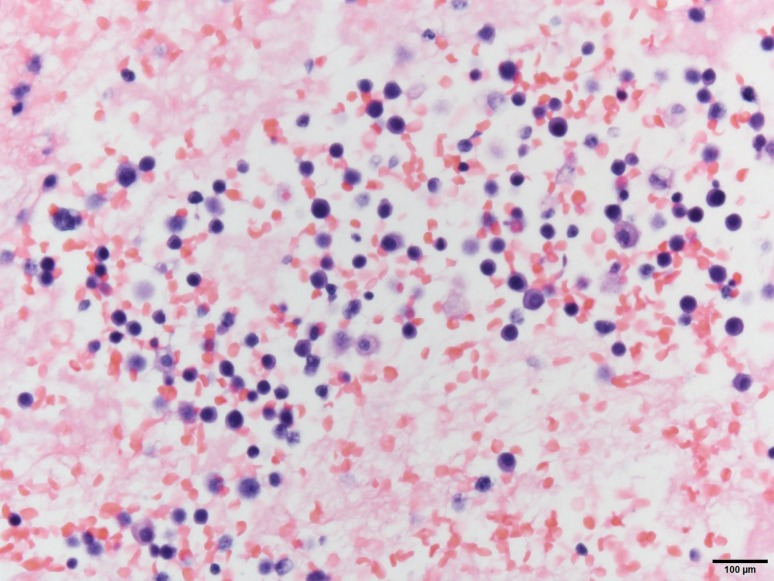
A cell block preparation of pleural fluid shows a prominent plasma cell concentration by hematoxylin and eosin staining

**Fig. 5:**
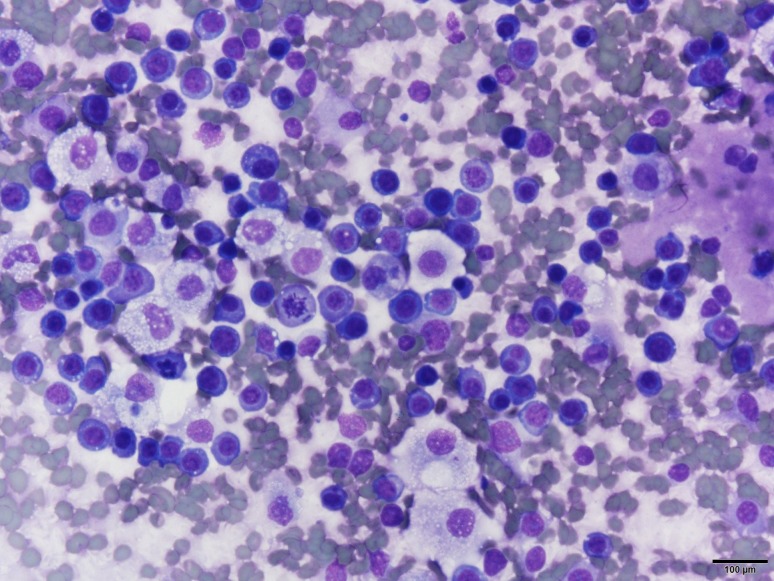
Numerous atypical plasma cells with increased mitotic activity by Diff Quik air-dried stained slide

**Fig. 6:**
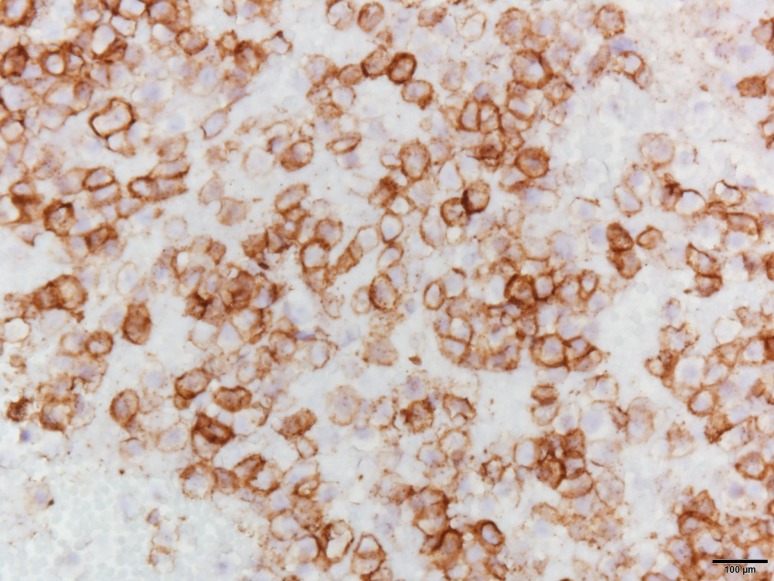
The CD138 immunostain highlights numerous clonal atypical plasma cells

**Fig. 7:**
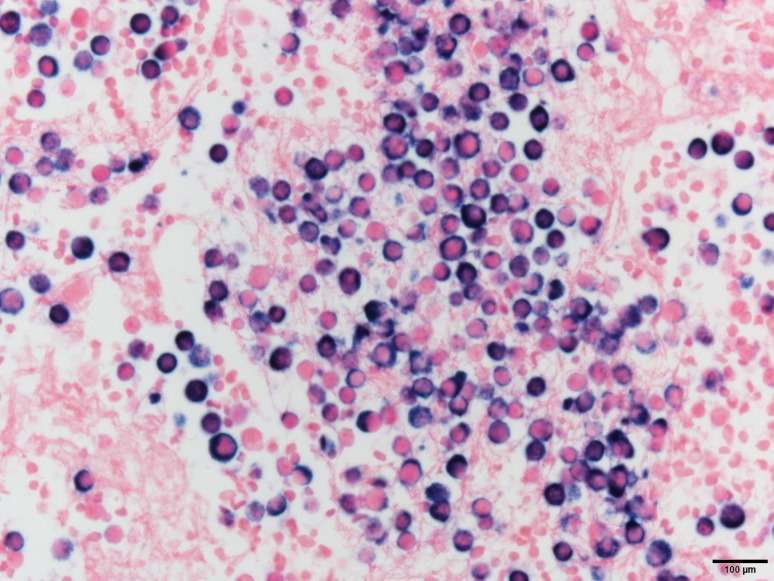
The kappa in situ hybridization stained slide shows that a majority of the CD138 plasma cells are positive for kappa light chains

He underwent thoracentesis with subsequent chest tube drainage demonstrating a serosanguineous appearing fluid and with approximately 700 mL output on water seal drainage during the first 24 h period. Chest X‑ray imaging post drainage showed diminished left lobe opacity (Fig. 3). Analysis of pleural fluid was undertaken with initial suspicion for infectious process and only modest consideration of recalcitrant MM. Pleural fluid immunohistochemical stains were performed (Figs. 4, 5, 6 and 7). CD138 immunostaining demonstrated atypical plasma cells with kappa restriction (Fig. 7). Complete kappa restriction was consistent with plasma cell dyscrasia and suggested involvement of the patient’s MM. The patient was informed that if dyspnea significantly increased prior to follow-up he should rapidly return to the oncology clinic. If rapid re-accumulation occurred, there was a discussion of repeat thoracentesis or placement of pigtail catheter and the option of placement of a PleurX catheter by interventional radiology. Pleurodesis was not felt to be a viable option but the patient expressed interest in PleurX catheter placement. Figs. 4, 5, 6 and 7. Pleural fluid immunohistochemical staining demonstrating plasma cell involvement of pleural effusion. Fluid was drained by thoracentesis.

The patient, readmitted with one week later with shortness of breath, was found to have re-accumulation of left pleural effusion with chest tube placed that initially drained 1800 ml. The patient had a PleurX catheter placed 3 days after admission. The patient was found to have worsening pain, dysphagia, chronic kidney disease stage II (CKD II), anemia, pancytopenia, and severe protein calorie malnutrition despite PleurX catheter intervention and ultimately the patient discussed discharge to comfort care with family and providers due to extreme weakness and inability to function at home. Unfortunately, the patient passed away two weeks later.

## Discussion

Multiple myeloma (MM) is a malignant proliferation of plasma cells that mainly affects the bone marrow. It is a common hematologic malignancy. Areas other than the bone marrow may be invaded as well, particularly the thorax resulting in pleural effusion, which affects about 6% of patients with MM [5]. A wide range of etiologic factors may cause pleural effusion in MM, including congestive heart failure due to hyperviscosity or amyloidosis, renal failure, pulmonary embolism, hypoalbuminemia and infections due to associated hypogammaglobulinemia and chemotherapeutic marrow depression, and rarely infiltration of tumor cells [6].

MPE is considered the end stage of the disease and may result from extension of adjacent plasmacytomas into the pleural space, or direct implantation into the pleura. Therefore, it is supposed that large quantities of immunoglobulins are secreted by malignant plasma cells in pleura, leading to high colloid osmotic pressure of the fluid to a degree that impairs normal absorption [7]. To our knowledge, this is the first case of MPE that was reported after multiple relapses of MM. The presence of MPE is a strong indicator of impending death and need for comfort care in case of multiple relapses; in our case mortality was less than 2 months of MPE onset. It was postulated that MPE usually develops during the treatment course of MM and is associated with an extremely poor prognosis, with a median reported survival of fewer than 4 months [8]. MPE represents clinical progression of the disease in itself and is associated with less mature population of plasma cells [9]. Furthermore, it was noted to coincide with other morbid complications such as diffuse bone loss [10] and pulmonary embolism [9].

With regard to the treatment regimen this patient followed and another plausible mechanism as well as clinical complexity to consider is the patient’s treatment with proteasome inhibitors such as carfilzomib. In 2012, this specific agent was approved for the treatment of relapsing MM. This is particularly relevant given the studied occurrence of pulmonary complications with some of the proteasome inhibitors. Data were analyzed on a large cohort of patients who participated in phase II oncarfilzomib studies [11]. In this study, there was documentation of pulmonary adverse events; most notably and predictably dyspnea occurred frequently in (42% of patients). Interestingly, pleural effusion occurred in 4% of patients. It is unclear whether these 4% of cases were causative but it can be presumed that some subfraction indeed was caused by administration of this agent. Given this not unsubstantial adverse event with this agent which may be a class effect to proteasome inhibitors, physicians should be considered whether this agent should be continued in the setting of MPE. Another drug in this class, bortezomib, has also been reported to have a high occurrence of pulmonary side effects. In one notable study of 13 Japanese MM patients on bortezomib, 4 patients developed pulmonary complications including pleural effusion. It was hypothesized that since this class interacts with NF-kB, there could potentially be a mechanism for the lung injury noted on autopsy of one patient [12]. Moreover, improvement was seen with corticosteroids. Of course, this is somewhat speculative but should give the studious clinician pause when confronting MPE in a patient with MM on proteasome inhibitors.

Lenalidomide plus dexamethasone for treatment of MM was studied thoroughly and showed significant improvement in survival [13]. In 2013, our patient was found to have recurrence of his MM with 50% bone marrow involvement. Therefore, the patient started on 25 mg lenalidomide and dexamethasone for 21 days for a total of thirteen cycles over the course of one year, which followed by bone marrow biopsy that showed 11–12% polyclonal plasma cells without definitive morphologic or immunophenotypic evidence of recurrent plasma cell myeloma, which indicated re-treatment success of lenalidomide therapy during the treatment course of our patient.

The diagnosis of myelomatous pleural effusion was confirmed by cytomorphologic analysis of the lymphoid cells in the pleural effusion, which revealed a large majority of dystrophic plasma cells, in addition to immunohistochemical staining. Table 1 demonstrates the three diagnostic criteria to confirm a myelomatous pleural effusion [14]. Patients can present in a variety of ways that are not typically thought to be consistent with MM. Thus, a high index of suspicion is required to diagnose patients on chemotherapy with myelomatous effusion, given its rarity and limited available information. An accurate diagnosis is essential for choosing the appropriate treatment. There is no standard treatment strategy for MPE and affected patients are usually resistant to treatment and often relapse. Various antimyeloma agents were used including VAD regime (vincristine, adriamycin, and dexamethasone), prednisolone, melphalan, etoposide, and cisplatin [15]. Some novel agents have been proposed. Bortezomib, a novel proteasome inhibitor which has been shown to overcome some of the poor prognostic factors in myeloma, has been approved and is effective in patients with refractory myeloma, but may itself damage the pulmonary system [12, 16].

**Table 1:** Diagnostic criteria of myelomatous pleural effusion

Demonstration of a monoclonal protein in pleural fluid electrophoresis
Detection of atypical plasma cells in the pleural fluid
Histologic confirmation with a pleural biopsy sample or by autopsy

In this report, we discussed a case of MM with multiple relapses that eventually developed MPE and mortality was less than 2 months after pleural effusion diagnosis.

## Conclusion

Pleural effusion carries a differential diagnosis which may include malignancy but is commonly thought to be less specific to multiple myeloma but should still remain in the differential diagnosis. To our knowledge, this is the first case of myelomatous pleural effusion (MPE) that was reported after multiple relapses of MM. MPE is a very rare complication of MM, and its presence is a strong indicator of imminent mortality and need for comfort care in case of multiple relapses. End-stage pleural effusion in MM in the setting of proteasome inhibitor adds more therapeutic and diagnostic challenges.

## Abbreviations

CKD II: Chronic kidney disease stage II
CT: Computed tomography
FISH: Fluorescence in situ hybridization
ISS: International staging system
LDH: Lactate dehydrogenase
MM: Multiple myeloma
MPE: Myelomatous pleural effusion
MRI: Magnetic resonance image
R-ISS: Revised international staging system
T: Thoracic spinal segment
